# Mutations in *Mll2*, an H3K4 Methyltransferase, Result in Insulin Resistance and Impaired Glucose Tolerance in Mice

**DOI:** 10.1371/journal.pone.0061870

**Published:** 2013-06-24

**Authors:** Michelle Goldsworthy, Nathan L. Absalom, David Schröter, Helen C. Matthews, Debora Bogani, Lee Moir, Anna Long, Christopher Church, Alison Hugill, Quentin M. Anstee, Rob Goldin, Mark Thursz, Florian Hollfelder, Roger D. Cox

**Affiliations:** 1 Medical Research Council (MRC) Harwell, Diabetes Group, Harwell Science and Innovation Campus, Oxfordshire, United Kingdom; 2 Department of Biochemistry, University of Cambridge, Cambridge, United Kingdom; 3 Department of Academic Medicine, St Mary's Hospital Campus, Imperial College London, United Kingdom; 4 Institute of Cellular Medicine, Newcastle University, The Medical School, Framlington Place, Newcastle-upon-Tyne, United Kingdom; 5 Department of Histopathology, Imperial College London, St Mary's Hospital Campus, London, United Kingdom; Consiglio Nazionale delle Ricerche, Italy

## Abstract

We employed a random mutagenesis approach to identify novel monogenic determinants of type 2 diabetes. Here we show that haplo-insufficiency of the histone methyltransferase myeloid-lineage leukemia (*Mll2/Wbp7*) gene causes type 2 diabetes in the mouse. We have shown that mice heterozygous for two separate mutations in the SET domain of *Mll2* or heterozygous *Mll2* knockout mice were hyperglycaemic, hyperinsulinaemic and developed non-alcoholic fatty liver disease. Consistent with previous *Mll2* knockout studies, mice homozygous for either ENU mutation (or compound heterozygotes) died during embryonic development at 9.5–14.5 days post coitum. Heterozygous deletion of *Mll2* induced in the adult mouse results in a normal phenotype suggesting that changes in chromatin methylation during development result in the adult phenotype. *Mll2* has been shown to regulate a small subset of genes, a number of which *Neurod1*, *Enpp1, Slc27a2*, and *Plcxd1* are downregulated in adult mutant mice. Our results demonstrate that histone H3K4 methyltransferase Mll2 is a component of the genetic regulation necessary for glucose homeostasis, resulting in a specific disease pattern linking chromatin modification with causes and progression of type 2 diabetes, providing a basis for its further understanding at the molecular level.

## Introduction

Type 2 diabetes is a major and increasing health problem worldwide. It is estimated that the global average prevalence of Diabetes worldwide is 10% (WHO World Health Statistics 2012 report). Type 2 diabetes is generally a later onset form of diabetes and is characterized by defects in insulin action and secretion. In addition to environmental factors, such as obesity, leading to increased diabetes risk it has been clearly demonstrated that there is a complex genetic component. In recent years there has been great success using genome wide association studies to identify, in humans, candidate loci containing genes conferring risk for type 2 diabetes or sub-diabetic traits, although in the context of these studies these are small effect alleles [1]–[14] (reviewed [15], [16]).

To maintain or initiate gene expression, the local chromatin structure must be in an active state to allow access to transcription factor complexes. Histone molecules have a range of acetylation or methylation modifications that are associated with active or inactive chromatin (reviewed in [17], [18]). Furthermore, transcriptional activity is often associated with trimethylation at the fourth lysine residue of histone H3 (H3K4) at active promoter regions [19], [20]. Intrauterine growth retardation (IUGR) induced in rats causes the *Pdx1* locus in pancreatic β-cells to undergo changes in histone methylation and acetylation that results in progressive transcriptional silencing and development of type 2 diabetes [21]. Similarly, adult *Glut4* gene transcription is reduced in skeletal muscle in an IUGR rat model due to changes in chromatin methylation and DNA methylation [22]. Finally, *Hnf4α*, a gene linked to diabetes, is progressively epigentically silenced in rat beta cells due to poor maternal diet and aging [23]. *In vitro* studies of human monocytes under normal or high glucose indicated changes in expression of candidate genes linked to glucose dependent changes in histone methylation [24]. These studies provide limited evidence that chromatin remodeling is involved in glucose homeostasis

Phenotype-driven N-ethyl-N-nitrosourea (ENU) mutagenesis screens have been shown to be an effective tool for the identification of novel murine models of human disease [25], [26] including new mouse models of type 2 diabetes [27], [28]. Using this approach, we identified a novel mouse model of type 2 diabetes that also exhibits features of non-alcoholic fatty liver disease (NAFLD). By mapping and sequencing we have identified a mutation in a histone 3 lysine 4 (H3K4) methyltransferase, myeloid-lineage leukemia 2 (*Mll2/Wbp7*). We also identified additional alleles in the mouse and confirmed the functional link between this gene and type 2 diabetes phenotypes [29]. Finally, we describe evidence that *Mll2* contributes to glucose homeostasis through altered gene regulation established during development.

## Materials and Methods

### Animal Husbandry

Mice were kept in accordance with UK Home Office Welfare guidelines and project license restrictions and in addition the study was approved by the local Ethical Review Panel committee. *Mll2*FC and *Mll2*KO^+/−^ mice were a kind gift from Professor Frances Stewart (Dresden University of technology, Germany). B6.Cg-Tg(UBC-cre/ERT2)1Ejb/J a Tamoxifen-inducible cre was obtained from the Jackson Laboratory (Bar harbor, ME). All mouse lines were maintained by on a C3H/HeH background by Backcrossing. Mice were sacrificed by either cervical dislocation or exsanguination under general anesthesia.

### Tamoxifen Knockout *Mll2*FC

Tamoxifen was resuspended in Corn Oil +2% Ethanol at a concentration of 30 mg/ml. Mice were dosed at 8 weeks of age via oral gavage with 200 mg/kg Tamoxifen or vehicle control once a day for 5 days.

### Intraperitoneal Glucose Tolerance test and biochemistry

Mice were tested using the EMPReSS IPGTT (http://empress.har.mrc.ac.uk). In insulin Tolerance tests (ITT), mice were fasted for 4 hours and a T0 blood sample taken and an Intraperitoneal injection of 2iU/Kg of insulin administered. Subsequent blood samples were taken at 15, 30, 45 and 60 minutes and blood glucose determined using a GM9 glucose analyser (Plasma, Analox, UK) or glucotrend strips (Whole blood). Plasma insulin was measured using a Mercodia ultra-sensitive mouse ELISA kit according to the manufacturer's instructions. The plasma concentrations of glucose, triglycerides, total cholesterol, HDL cholesterol and LDL cholesterol were measured on an AU400 (Olympus UK).

### Isolated Islets

Mice were killed by cervical dislocation, the pancreas removed, and islets isolated by liberase digestion and handpicking [30]. Cells were maintained in this medium at 37°C in a humidified atmosphere at 5% CO_2_ in air, and used 24 hours after the isolation. Insulin secretion was measured during 1-hour static incubations in Krebs-Ringer Buffer as described previously [30].

### DNA archive screen

The Harwell DNA archive was screened utilising High-resolution DNA melting analysis performed on the Idaho Technology LightScanner as described previously [30].

### Genotyping and sequencing

Genomic DNA was extracted from either mouse tail or ear tissue using a Qiagen DNeasy tissue kit (Qiagen, UK) according to the manufacturer's instructions. The DNA of the founder F1 mouse was sequenced within coding regions of 26 of the 27 genes within the mapped interval, not including the *Gapdhs* gene that was also sequenced apart from 150 bp of repeat sequence (Table S1). Genotyping of the two *Mll2* ENU mutations was performed by pyrosequencing. The *Mll2* KO mice were genotyped using a generic Neo PCR assay.

### Histology and Islet immunohistochemistry

The pancreas and liver from each mouse was fixed in neutral buffered formaldehyde and mounted in wax longitudinally. Serial sections were cut and stained with Hematoxylin and Eosin (H&E). A rabbit ABC staining system (Santa Cruz Biotechnology) was used according to manufacturer's instructions. The following primary antibodies were used: rabbit anti-human glucagon (1∶40; AbD Serotec), rabbit anti-somatostatin (2 µg/ml; Chemicon International). Sections were counterstained with Gill's formulation no. 2 hematoxylin. H-E–stained sections from each mouse were photographed completely, and islet area calculated using Adobe Photoshop to measure islet area and total pancreas section area in each image. Liver histology was assessed by an expert histopathologist (RG) for steatosis and steatohepatitis using the NIDDK NASH Clinical Research Network histological classification [31].

### Embryo Dissection

Mice heterozygous for both ENU mutations were crossed to mice heterozygous for the same mutation or the *Mll2* KO allele. Embryo dissections were carried out between 8.5 and 14.5 days *post coitum (dpc*) and the embryonic phenotype was visually analyzed.

### Quantitative RT-PCR

RNA was extracted from snap frozen tissues with RNeasy Mini Prep (Qiagen) kits according to the manufactures instructions. cDNA generated by Superscript II enzyme (Invitrogen, UK) was analysed by quantitative RT-PCR using the TaqMan system based on real-time detection of accumulated fluorescence (ABI Prism 7700, Perkin-Elmer Inc., USA). Gene expression was normalised relative to the expression of glyceraldehyde-3-phosphate dehydrogenase *(Gapdh)*. Taqman Probes with FAM tags were purchased from Applied Biosystems (ABI, USA). Samples were tested in triplicate and results expressed relative to *Gapdh*.

### Site-directed mutagenesis and Protein expression

The SET domain from *MLL1* comprising the last 178 amino acids (with 86% sequence identity to the MLL2 SET domain sequence, Figure S1) was expressed as a glutathione S-transferase (GST) fusion protein from the expression vector pGEX-2T (GE Healthcare) in *Escherichia coli* Rosetta (DE3) (Novagen). (Several Mll2 constructs failed to express under identical conditions.) Expression was induced with 0.2 mM isopropyl- D-thiogalactopyranoside for 24 hours at 20°C. The enzyme was purified over a glutathione-Sepharose 4B column (GE Healthcare). After dialysis against a buffer containing 50 mM Tris/HCl, pH 8.5, 100 mM NaCl, 1 mM dithiothreitol, and 10% glycerol the enzyme was stored at −80°C. The enzyme concentration was determined using the FluroProfile® Protein Quantification kit (Sigma-Aldrich). The expression and purification procedure of the M3884K mutant of *MLL1* was identical to the wild-type enzyme.

### *In vitro* methylation assays

The biotinylated peptide substrates comprising the first 21 N-terminal amino acids of human histone H3 were immobilized on streptavidin-coated 96 well microtiter plates (Sigma) by incubating 50 µL of 5 µg/mL peptide in PBS per well for 1 h at room temperature. After washing three times with water, each well was incubated with 200 µL of 3% bovine serum albumin in PBS for 2 h at room temperature. Following a washing step with PBS (3 times) each well was incubated with 50 µl of methylation reaction mixture (1 µM methyltransferase, 1 mM *S*-adenosyl-L-methionine (Fluka; purity ≥80%, stored in 10 mM H_2_SO_4_ at −20°C), 50 mM Tris-Cl, pH 8.5, 100 mM NaCl, 2 mM dithiothreitol) or the control reaction (1 mM *S*-adenosyl-L-methionine, 50 mM Tris-Cl, pH 8.5, 100 mM NaCl, 2 mM dithiothreitol). The plates were incubated for the indicated time intervals at 30°C and subsequently washed three times with PBS. To detect the respective modifications each well was incubated with 50 µl of anti-H3K4Me1, anti-H3K4Me2, or anti-H3K4Me3 antibody (diluted 1∶250 in 3% bovine serum albumin, PBS) for 45 min at room temperature. The wells were then washed twice with PBST (PBS-0.5% Tween-20)/500 mM NaCl followed by washing twice with PBST and 3 times with PBS. The wells were then incubated with 50 µL of the secondary anti-rabbit IgG horseradish peroxidase conjugate (Sigma-Aldrich) (diluted 1∶5000 in 3% bovine serum albumin, PBS) for 45 min at room temperature. After a washing step with PBST (three times) and with water (three times) each well was incubated with 100 µl of TMB Microwell Horseradish Peroxidase Substrate (KPL) for 10 min. 50 µl of 1M H_3_PO_4_ were added and the absorbance measured at 450 nm on a SpectraMaxPlus reader (Molecular Devices). For each peptide substrate, the values obtained without enzyme were defined as background and subtracted from those obtained for the enzymatic methylation. The synthetic peptides used for the methylation assays correspond to amino acids 1–21 of human histone H3 with lysine4 unmodified, monomethylated or dimethylated followed by a GG linker and biotinylated lysine (Upstate).

### Statistical Analysis

The calculations and statistical analyses (2-sample t-test were conducted using Excel and Prism). Unless otherwise specified data is expressed as mean ± SD. Differences with a p<0.05 were defined as significant.

## Results

### Identification of a mouse hyperglycaemia model

A random phenotype driven N-ethyl-N-nitrosourea (ENU) mutagenesis screen, where BALB/c male mice were treated with ENU and then crossed to female C3H/HeH mice was performed as previously described [25]. The *Mll2* line was identified as an individual G1 (BALB/c x C3H/HeH) male mouse (called GENA263) with elevated free-fed blood glucose [32]. Inheritance of the phenotype was confirmed by generating offspring by backcrossing to C3H/HeH mice and measuring glucose tolerance at 12 weeks of age in an intraperitoneal glucose tolerance test (IPGTT, data not shown).

### Identification of mutations in *Mll2*

**Figure 1:**
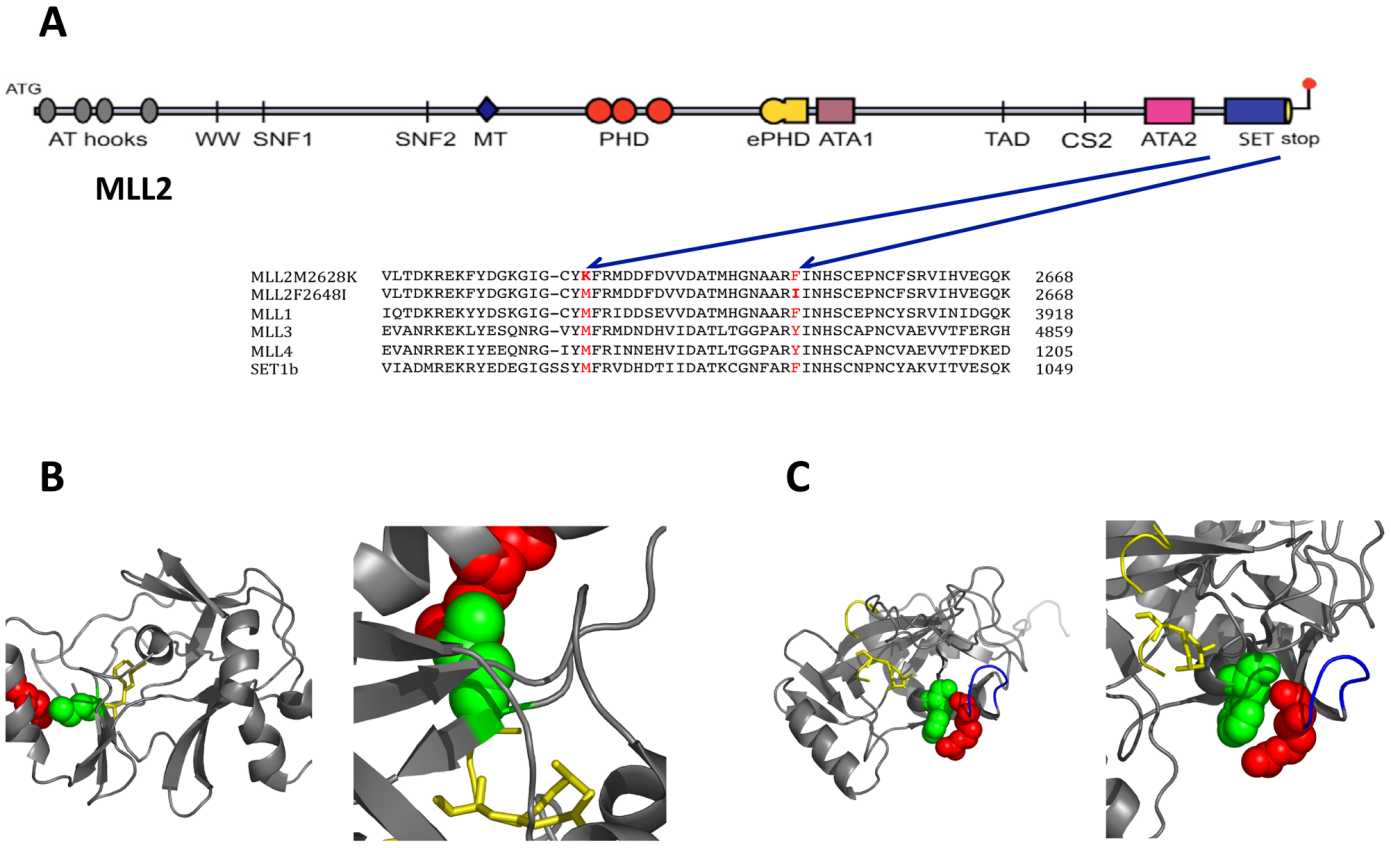
Position of ENU induced mutations within the SET domain of *Mll2*. ***A***: Schematic depicting the functional domains within the *Mll2* gene. The amino acid sequence alignment for the murine MLL-family of proteins in the SET domain is shown indication the positions of the 2 ENU mutations. ***B***: The location of the methionine to lysine mutation (green) within the binding groove of MLL2 the lysine of the histone is shown as yellow stick. **C**: The location of the phenylalanine to iosleucine mutation (red) in the 1^st^ alpha helix of the SET-C domain.

To identify the underlying mutation, mutant mice were backcrossed to C3H/HeH for two generations, phenotyped by an IPGTT and placed into affected or unaffected groups where affected mice had glucose levels 2 standard deviations (SD) or more above the population mean (data not shown). A genome-wide scan was performed on DNA from these crosses and a region of BALB/c DNA identified on chromosome 7 between D7Mit267 and D7Mit25 that was associated with the impaired glucose tolerance phenotype. Successive backcrossing to C3H/HeH mice and genotyping of DNA recombination events narrowed this candidate region to a 350 kilo-base-pair (kbp) region of chromosome 7. All the coding regions of 26 of the 27 genes within the region, not including spermatogenic *Gapdh*, (Table S1) were sequenced in the founder F1 DNA to identify heterozygous base pairs. An ENU induced thymine to adenine (T7883A, transcript Wbp7-001, ENSMUST00000108154, NCBIM37) transversion was identified in exon 36 of the gene encoding myeloid-lineage leukemia 2 (*Mll2*/*Wbp7*) (NM_029274, NP_083550). The Mll2T7883A mutation causes a methionine to lysine amino acid change at residue 2628 (M2628K) within the highly conserved SET (Su(var)3-9, enhancer(zeste)) methyltransferase domain (Figure 1A) [33]. As the crystal structure of the homologous MLL1 SET domain has been elucidated it is possible to map the *Mll2* ^M2628K^ mutation onto this structure where it corresponds to residue MLL1^M3884^ and occupies a position in the lysine binding groove (Figure 1B) [34].

To probe whether the observed phenotypes can be ascribed to an impairment of the methyltransferase function of the SET domain, a M3884K mutant MLL1 (the SET domains are highly conserved between the two genes, Figure S1) equivalent to M2628K in MLL2 (that in our hands could not be expressed) was tested in *in vitro* assays (Figure S2). The SET domain of wild-type and mutant MLL1 was thus expressed in *E*.coli, purified by affinity chromatography and histone mono-, di- and trimethylation was quantified by ELISA (Figure S3) [35]. The MLL1^M3884K^ protein had significantly reduced methyltransferase activity compared to the MLL1 wild type, demonstrating this residue was indeed required for methyltransferase activity (Figure S2).

In order to confirm that mutation of *Mll2* underlies the phenotypes we sought to identify additional mutant alleles of *Mll2*. Firstly, we screened for mutations within the *Mll2* SET methyltransferase domain using the Harwell ENU DNA/Sperm archive [29], [36]. An additional thymine to adenine (T7942A) transversion was identified and results in a phenylalanine to isoleucine amino acid change at residue 2648 (NP_083550, F2648I). The *Mll2* ^F2648I^ mutation maps to MLL1^F3904^ and is located in an α5 helix of the SET-C domain in a region of the protein important for interaction with the co-factor *AdoHcy* (Figure 1C) [34]. Secondly, a global knockout of the *Mll2* allele was kindly provided by Professor Frances Stewart (Dresden University of Technology, Germany) for additional analysis [33].

### Homozygous *Mll2* mutants are embryonic lethal

**Figure 2:**
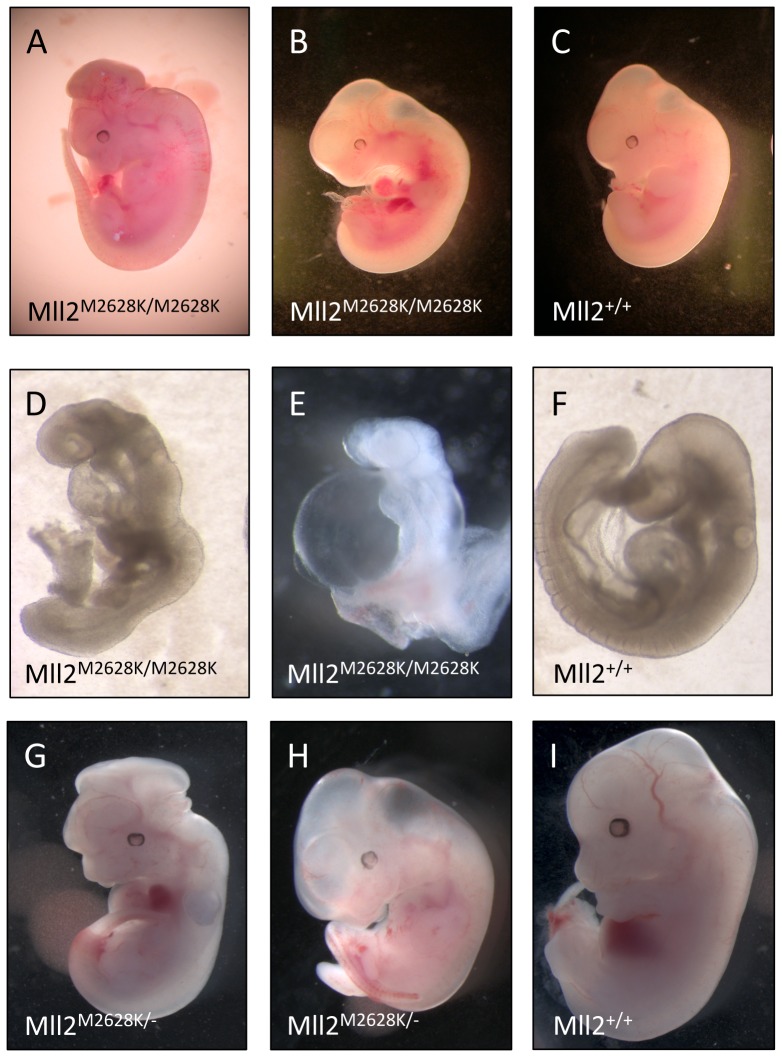
*Mll2 ^M2628K/M2628K^* has an identical embryonic lethal phenotype to *Mll2 ^M2628K/−^*. ***A***: *Mll2^ M2628K/M2628K^* embryo with exencephaly. ***B***: *Mll2^ M2628K/M2628K^* littermate exhibiting pericardial oedema and ***C***: 12.5 dpc *Mll2^+/+^* embryo. ***D***: *Mll2* ^M2628K/M2628K^ littermate showing abnormal heart looping, growth retardation and impaired turning. ***E***: 9.5 dpc *Mll2^ M2628K/M2628K^* embryo detail showing severe anterior truncation, abnormal heart looping and severe pericaridial oedema and ***F***: 9.5 dpc *Mll2^+/+^* embryo. ***G***: and ***H***: 12.5 dpc *Mll2 ^M2628K/−^* trans heterozygous littermates with exencephaly and generalised oedema. ***I***: 12.5dpc *Mll2^+/+^* embryo.

Homozygous *Mll2* knockout mice die of widespread apoptosis prior to 11.5 dpc and we therefore tested the functionality of our 2 ENU mutations in homozygous individuals [33]. No homozygous pups were identified from these matings (Table 1) showing that homozygosity of both the ENU alleles is non-viable. To determine the time and cause of lethality, we dissected pregnant females at 8.5, 12.5 and 14.5 dpc and genotyped the embryos for the *Mll2* ^M2628K/M2628K^ allele. The percentage of homozygous individuals was lower than expected at both 12.5 and 14.5dpc (Table 1). This suggested that the majority of M*ll2* ^M2628K/M2628K^ embryos were dying between 8.5 and 11.5 dpc. The most common defects identified in the embryos were evident as early as 10.5 dpc and up to 12.5 dpc and included pericardial effusion, abnormal heart looping, exencephaly and various head abnormalities and anterior truncation defects (Figure 2A–F). The observed heart and/or circulation defects are the most likely cause of non-viabilty in *Mll2* ^M2628K/M2628K^ embryos.

**Table 1:** Loss of homozygous *Mll2^M2628K^* embryos during *in utero* development.

Days post-coitum	Genotype		
	*Mll2^+/+^*	*Mll2^M2628K/+^*	*Mll2^M2628K/M2628K^*
8.5	13 (10.75)	22 (21.5)	8 (10.75)
12.5	12 (12)	32 (24)	4 (12)
14.5	5 (6)	20 (12)	1 (6)
birth	10 (6.25)	15 (13)	0 (6.25)

Number of embryos and expected number according to Mendelian inheritance shown in brackets.

As the homozygous lethal phenotype of *Mll2* ^M2628K/M2628K^ is not identical to the published phenotype of *Mll2* knockout mice we investigated the effect of combining the two mutations. We set up crosses between *Mll2* ^M2628K/+^ and heterozygous *Mll2^+/−^* knockout mice to create *trans*-heterozygote *Mll2* ^M2628K/−^ animals. No *trans*-heterozygous pups were born (Table 2). Dissection of pregnant females at 8.5, 12.5 and 14.5 dpc and determined that the embryos were dying between 8.5 and 12.5 dpc. The defects observed in these *trans*-heterozygotes were similar to *Mll2* ^M2628K/M2628K^ embryos and included exencephaly, oedema and pericardial effusion (Figure 2G–I). Thus the two mutations fail to complement.

**Table 2:** Non-complementation of the *Mll2^M2628K^* and *Mll2* knockout alleles; loss of compound heterozygous embryos during *in utero* development.

Days post-coitum	Genotype			
	*Mll2^+/+^*	*Mll2^M2628K/+^*	*Mll2^+/−^*	*Mll2^M2628K/−^*
9.5	6 (4)	2 (4)	4 (4)	4 (4)
12.5	13 (11)	13 (11)	9 (11)	9 (11)
14.5	2 (3)	3 (3)	7 (3)	0 (3)
birth	5 (6.5)	9 (6.5)	12 (6.5)	0 (6.5)

Number of embryos and expected number according to Mendelian inheritance shown in brackets.

### Mutation of *Mll2* leads to adult impaired glucose tolerance and insulin resistance

**Figure 3:**
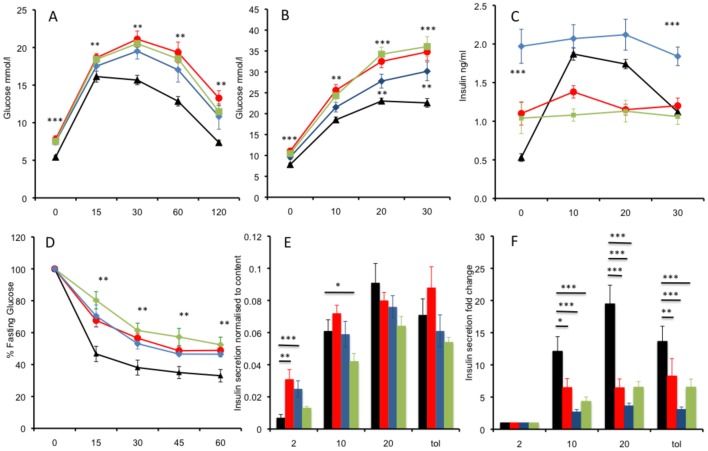
Mutation of *Mll2* leads to impaired glucose tolerance and insulin resistance and impaired insulin secretion in isolated Islets. **A**: Plasma glucose measured in an intraperitoneal glucose tolerance test in male mice at 12 weeks of age, animals heterozygous for *Mll2^M2628K/+^* (red circles N = 20), *Mll2^F2648I/+^* (blue diamonds N = 7) and the *Mll2^+/−^* Knockout (green squares N = 16) show impaired glucose tolerance compared to wild-type littermates (black triangles N = 22) **B**: Plasma glucose measured during a 30 minute IPGTT in male mice at 16 weeks of age, animals heterozygous for the *Mll2^M2628K/+^* (red circles N = 14), *Mll2^F2648I/+^* (blue diamonds N = 7) and the *Mll2^+/−^* Knockout (green squares N = 12) show impaired glucose tolerance compared to wild-type littermates (black triangles N = 25) **C**: Plasma insulin measured during a 30 minute IPGTT in male mice at 16 weeks of age, animals heterozygous for *Mll2^M2628K/+^* (red circles N = 14), *Mll2^F2648I/+^* (blue diamonds N = 7) and the *Mll2^+/−^* Knockout (green squares N = 12) exhibit fasting hyperglycaemia and fail to secret insulin in response to a glucose challenge in comparison to wild-type littermates (black triangle N = 25). **D**: Insulin tolerance tests carried out on male mice at 12 weeks of age. Data was normalized for differences in fasting glucose levels, response to insulin load was reduced in animals heterozygous for *Mll2^M2628K/+^* (red circles N = 9), *Mll2^F2648I^* (blue diamonds N = 7) and the *Mll2^+/−^* Knockout (green squares N = 10) compared to wild-type littermates (black triangles N = 9). **E**: Insulin secretion from islets isolated from *Mll2^M2628K/+^* (red bars), *Mll2^F2648I/+^* (blue bars) *Mll2^+/−^* Knockout (green bars) and wild-type littermates (black bars) in response to glucose (2, 10, 20 mM or tolbutamide (tol, 200 mM+2 mM Glucose)). Both the *Mll2^M2628K/+^* and *Mll2^F2648I/+^* Islets hypersecrete insulin at 2 mM glucose, insulin was elevated in the *Mll2^+/−^* islets but this failed to reach significance. Islets were isolated from 5 mice of each genotype. **F**: Insulin secretion data expressed as fold change. The data represent the mean of 5 animals (with 4 technical replicates per animal of 5 islets). All data are presented as Mean ± SEM, * p<0.05, **p<0.01, ***p<0.001, pairwise comparison student's t-test (compared to wt littermates).

Having established the underlying genetics, both ENU derived mutant alleles and global heterozygous knockout *Mll2* mice were further characterised for glucose tolerance and insulin sensitivity. Intraperitoneal Glucose Tolerance Tests (IPGTT) were carried out at 12 weeks of age (Figure 3A). In all 3 lines, in comparison to wild-type littermates, we observed significantly elevated plasma glucose at fasting and at all time points during the IPGTT, with levels failing to return to normal at 120 minutes. In order to further investigate the underlying physiological defect in the *Mll2* mutants we measured plasma glucose and insulin at 0, 10, 20 and 30 minutes after an intraperitoneal (IP) injection of glucose at 16 weeks of age (Figure 3B and C). The fasting plasma insulin concentrations of all 3 lines were significantly higher than the wild-type littermates. However unlike wild-type littermates *Mll2* mutant or heterozygous knockout mice showed a marked decrease in insulin secretion in response to the glucose challenge, suggesting that the impaired glucose tolerance observed is due in part due to an insulin secretory defect.

To test whether the mice are also insulin resistant Insulin Tolerance Tests (ITT) were carried out on new cohorts of mice at 12 weeks of age. An insulin load of 2IU/Kg was administered IP after a four hour fast and plasma glucose concentrations measured at 15, 30, 45 and 60 minutes post insulin injection. Results were normalised for differences in fasted glucose levels by expressing glucose concentrations as a percentage of the T0 concentration. There was a significant reduction in the fall of glucose levels in *Mll2* mutant and knockout mice in response to insulin (Figure 3D).

To investigate the failure of *Mll2* mutant mice to mount an appropriate insulin response to a glucose challenge, islets were isolated from 19 week old mice and insulin secretion assayed. The observed insulin secretion differed slightly in the two mutant ENU alleles compared to the heterozygous global knockout. All 3 alleles secreted insulin in response to increasing glucose concentrations and to the tolbutamide treatment control (Figure 3E). Both *Mll2* ^M2628K/+^ *and Mll2* ^F2648I/+^ alleles secreted significantly more insulin at 2 mM glucose, resulting in a lower fold change difference in secretion at 10 and 20 mM, which would appear to mirror the lack of increased insulin secretion observed in the whole animal studies (Figure 3F). Islets from mice heterozygous for the global *Mll2* knockout also secreted more insulin at 2 mM glucose although this did not reach statistical significance, however they secreted significantly less insulin at 10 mM glucose compared to wild-type littermate controls and had lower fold change differences at 10 and 20 mM glucose. Data from isolated islets is consistent with whole animal data; fasting hyperinsulinaemia and greatly reduced fold increase secretion of insulin in response to a glucose challenge.

### Tamoxifen induced Knockdown of *Mll2* in adult mice does not result in a glucose phenotype

**Figure 4:**
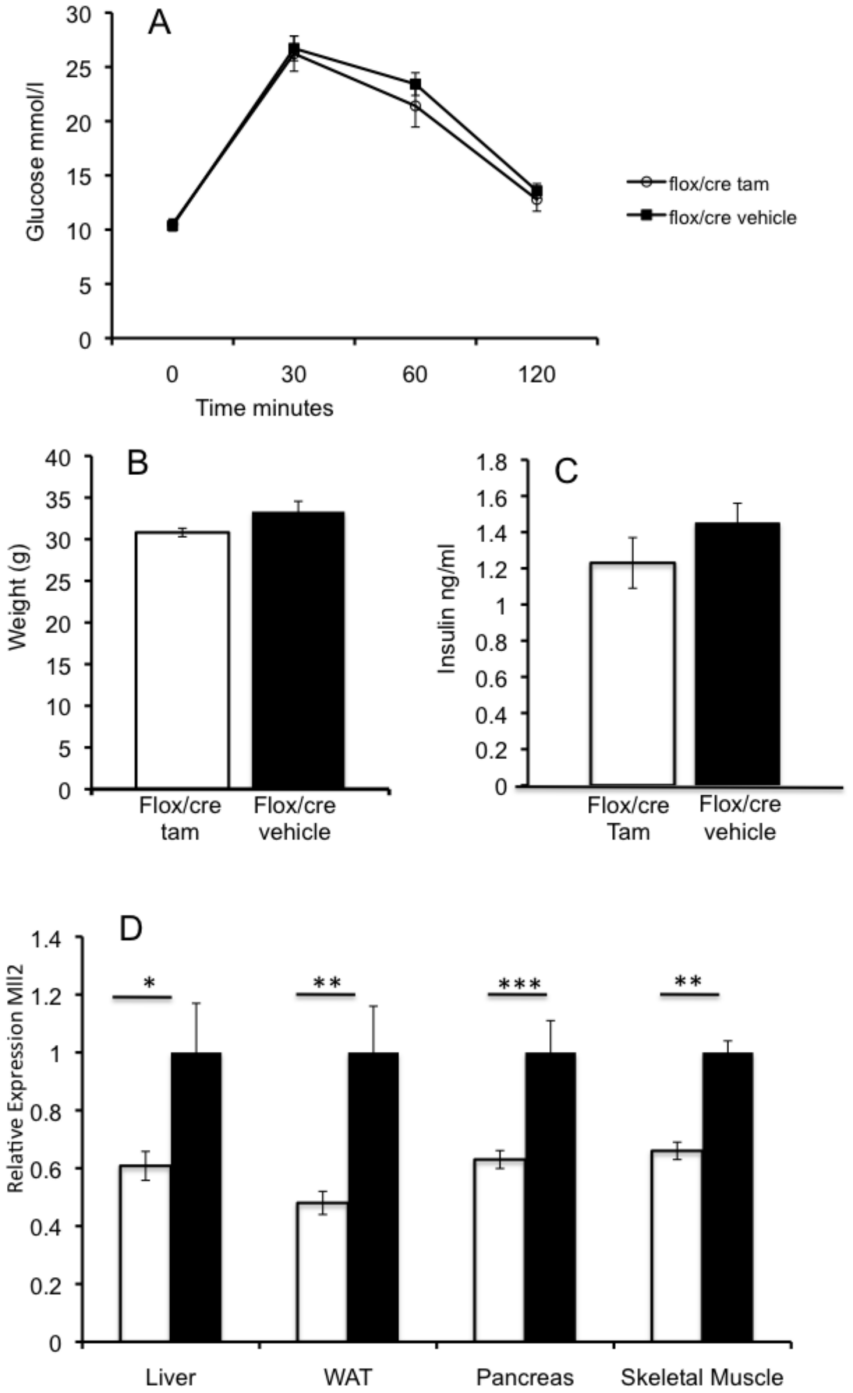
Adult-induced Knockout of *Mll2* does not result in a glucose phenotype. **A**: Plasma glucose measured in an IPGTT in male mice at 12 weeks of age, *Mll2* ^flox/+^/tamoxifen^cre/+^ Tamoxifen treated (N = 11) open circles, *Mll2* ^flox/+^/tamoxifen^cre/+^ Vehicle treated (N = 10) black squares. **B**: Weight at 12 weeks of age, **C**: Fasted insulin at 12 weeks of age **D**: Relative expression of *Mll2* gene after tamoxifen treatment in Liver, WAT, Pancreas and Skeletal muscle, mean of 8 biological replicates *Mll2* ^flox/+^/tamoxifen^cre/+^ Tamoxifen (open bars) vs *Mll2* ^flox/+^/tamoxifen^cre/+^ Vehicle (black bars) treated. Mean ± SEM, * p<0.05, **p<0.01, ***p<0.001, student's t-test.

In order to test whether the observed adult glucose phenotype was the result of early developmental effects or changes in the adult animal we carried out a heterozygous adult-induced knockout. A floxed *Mll2* allele was crossed with a tamoxifen-inducible ubiquitin-Cre and mice of all genotype classes treated with tamoxifen or vehicle at 8 weeks of age, in order to generate a knockout allele. Mice were phenotyped at 12 weeks using an IPGTT test. No significant difference was seen in either glucose tolerance, weight or fasted insulin in heterozygous mice (Figure 4A, B and C and Figure S4). Levels of *Mll*2 knockdown in tamoxifen treated mice was assayed by quantitative RTPCR (Figure 4D) and 41% reduction in *Mll2* levels was observed.

### Biochemical and histological analysis of *Mll2* mutants; evidence of fatty liver disease

**Figure 5:**
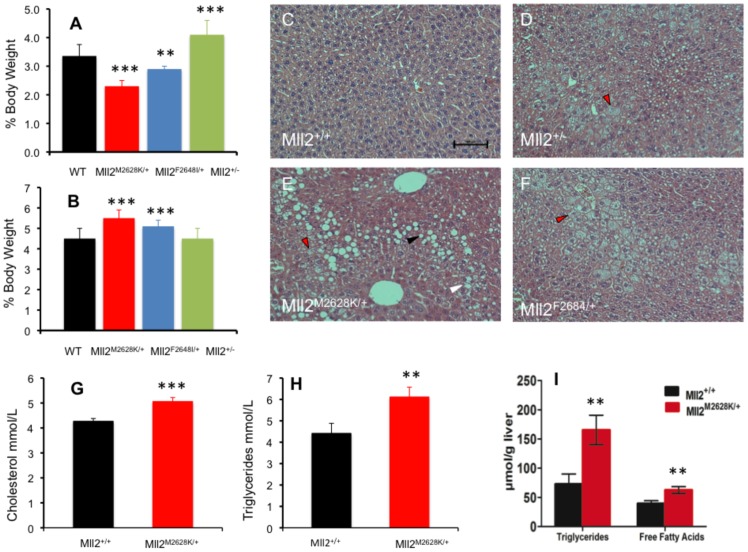
Biochemical and Histological analysis of *Mll2* mutants; NAFLD and dyslipidaemia. Cohorts of mice were culled at 19 weeks of age. **A**: Epididymal fat pad weights normalized for body weight, *Mll2^M2628K/+^* (N = 10) and *Mll2^F2648I/+^* (N = 7) cohorts exhibited abnormal peripheral fat deposition with reduced fat pads compared to wild-type littermate (N = 23) or *Mll2^+/−^* (N = 5). **B**: Liver weight normalized for body weight, *Mll2^M2628K/+^* (N = 10) and *Mll2^F2648I/+^* (N = 7) cohorts show hepatomegaly. **C–D**: Histological analysis of H&E stained liver sections demonstrated features consistent with mild NAFLD in all *Mll2* mutant and knockout lines (Figure 6C, D & E) with significant increases in macrovesicular steatosis (black arrow), microvesicular steatosis (red arrows) and ballooning hepatocyte degeneration (white arrow), compared to wild-type littermates. Biochemical analysis of plasma showed elevated Cholesterol (**G**) and Triglycerides (**H**). **I**: The steatosis was confirmed biochemically as liver triglycerides and liver free fatty acids were significantly increased in the M2628K mutation. The data represent the mean of 6 animals of each genotype class ±SEM * p<0.05, **p<0.01, ***p<0.001, student's t-test.

As these mice were insulin resistant we further investigated them for other metabolic disturbances. At 19 weeks of age mice were fasted for four hours then sacrificed and metabolic tissues were collected, additionally epididymal white fat pads and livers were weighed. The two mutant ENU alleles of *Mll2* exhibited dyslipidaemia and epididymal fat pads were significantly lighter at 19 weeks of age in the mutants versus wild-type littermates (Figure 5A). They also showed significant hepatomegaly compared to wild-type littermates (Figure 5B). There was no significant increase in body mass observed in the 2 ENU mutants or *Mll2* ^+/−^ knockout compared to wild-type littermates. Body composition of the *Mll2* ^M2628K/+^ mutant allele was additionally measured by DEXA analysis at 8, 12 and 18 weeks of age (Figure S5) with a brief increase in percentage fat mass observed at 12 weeks of age in the mutant (31.04±4.25% vs 26.85±4.87%). Histological analysis of liver sections demonstrated features consistent with NAFLD (Figure 5C–F). The severity was formally assessed using the semi-quantitative NIDDK histological score [31] and revealed significant increases in steatosis and ballooning hepatocyte degeneration compared to wild-type littermates. Biochemical analysis of plasma samples showed significantly increased cholesterol and triglyceride (Figure 5G and H). Biochemical analysis of liver tissue showed increased triglyceride and free fatty acid content in *Mll2* ^M2628K/+^ compared to wild-type littermates (Figure 5I).

### Histological analysis of Pancreatic Islets

Pancreatic sections from *Mll2* ^M2628K/+^ animals and wild-type littermates were examined for differences in both islet mass and architecture, 3 sections for 8 mice of each genotype class were examined. There was small significant increase (p<0.01) in islet area in heterozygous *Mll2* ^M2628K/+^ mice (1.237±0.077%) compared to wild-type litter mates (1.002±0.358%) in their percentage of islet areas (the percentage of a histological section identified as islet Table S2). Histological staining showed no qualitative difference in islet architecture in terms of arrangement or relative numbers of α- or δ-cells (data not shown)

### Altered gene expression in *Mll2* mutants

**Figure 6:**
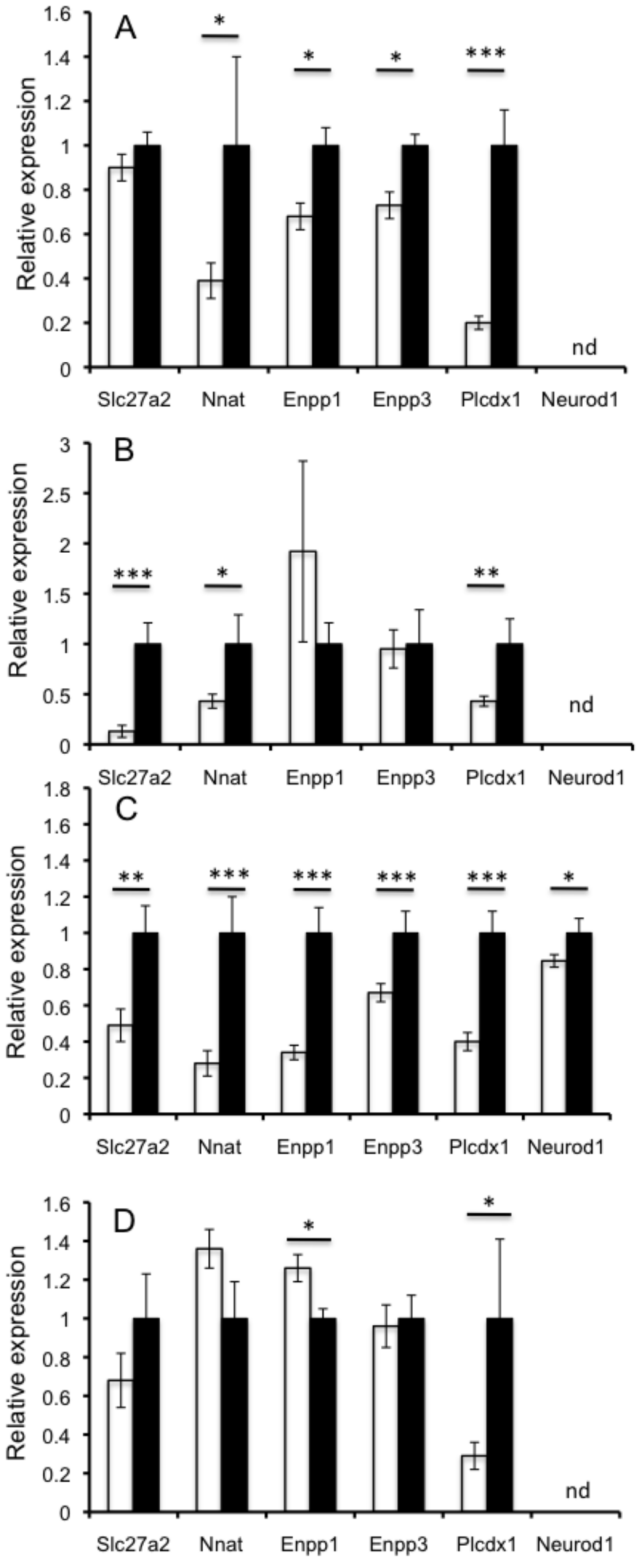
Relative expression of *Mll2* regulated genes. **A**: Liver, **B**: Epididymal White adipose, **C**: Isolated islets, **D**: Skeletal Muscle. Data represents 8 biological replicates, *Mll2^M2628K^*/+ (open bars) vs wt littermates, (black bars) data normalized to GAPDH. Relative expression ±SEM * p<0.05, **p<0.01, ***p<0.001, student's t-test. nd = not detectable.

The H3K4 methyltransferase Mll2 is thought to function briefly during development to alter cellular gene expression programmes that are then maintained by other redundant mechanisms [37]. This is consistent with our results as the adult knockout suggests that the glucose phenotype arises from changes in gene expression set during earlier development. We therefore decided to examine the expression of selected genes with links to glucose homeostasis and identified as altered in expression in published ES cell gene expression experiments using Mll2 mutants (see additional file 1 in [37]). These included *Slc27a, Enpp3* (and additionally *Enpp1* as it has been implicated in diabetes and is adjacent to its paralog *Enpp3* in the genome), *Plcxd1, Neurod1* and several genes downstream of *Neurod1*. The expression profile of these downregulated genes was investigated in adult *Mll2^M2628K^* ^/+^ heterozygous mutants in the metabolically important tissues liver, white adipose tissue, isolated islets and skeletal muscle (Figure 6A–D).

*Neurod1* is an important transcriptional regulator both in the developing pancreas and the mature beta cell. Its expression was significantly downregulated in mutant islets, therefore the relative expression of a number of genes regulated by *Neurod1* in the beta cell were examined. *Ins1* but not *Ins2* insulin gene expression was slightly upregulated, and both *Glucagon* and *Nnat* downregulated (Figure S6). *Nnat* expression was significantly reduced in liver and white adipose tissue (Figure 6A and B). *Slc27a2* expression was significantly reduced in islets and adipose tissue. There were no significant difference in liver or muscle (although a trend to reduction in the latter) (Figure 6 A–D). *Enpp1 and Enpp3* were significantly reduced in liver and islets, however expression was not altered in white adipose. However, *Enpp1* showed an increase in skeletal muscle and *Enpp3* showed no difference (Figure 6D). Phosphatidylinositol-specific phospholipase C, X domain containing 1 (*Plcxd1*) expression was reduced in all tissues examined.

## Discussion

We have identified two mutations in the histone methyltransferase *Mll2*, within the highly conserved carboxyl-terminal SET domain which is required for methyltransferase activity. Overlaying the two mutations on the recently determined crystal structure of its sister gene *MLL1* places the MLL2^M2628K^ mutation within the active site and the MLL2^F2628I^ mutation within the α5 helix of the SET-C domain [34]. Both mutations are embryonic lethal when homozygous or when crossed to a global *Mll2* knockout as expected, confirming both mutant alleles are nulls and abolish methyltransferase activity. This loss of activity has been further illustrated *in vitro* by the substitution of the mutant MLL2^M2628K^ amino acid into the highly conserved sister gene *MLL1*. When the mutation is introduced into the SET-domain of *MLL1*, there is impaired mono-methylation of H3K4 *in vitro*.

Haploinsufficiency of *Mll2* results in hyperglycaemia and hyperinsulinaemia at fasting and impaired glucose tolerance with blunted insulin secretion in response to a glucose load. Insulin tolerance tests further showed peripheral insulin resistance, with a reduced fold change in insulin secretion observed in isolated islets. Mice have no increase in body mass and mostly no difference in body composition. However, they exhibit abnormal plasma triglycerides, total cholesterol and reduced fat pad mass with concurrent hepatomegaly and increased hepatic fat accumulation when culled at 19 weeks of age.

NAFLD is a complex genetic trait strongly associated with type 2 diabetes and insulin resistance and is increasingly recognised as the leading cause for liver dysfunction and cirrhosis in the non-alcoholic, viral hepatitis negative population in Europe and North America [38]–[40]. The NAFLD phenotype spontaneously develops in *Mll2* mutants and may be in response to insulin resistance or alternatively may cause a pre-disposition to NAFLD.

As methylated H3K4 is associated with active chromatin, reduced MLL2 protein levels or H3K4 methyltransferase function could lead to changes in transcription of genes. A published inducible knockout of *Mll2* used expression profiling of ES cells to show that only a single developmental gene, *Magoh2*, is entirely dependent upon *Mll2* for its expression [37]. Knockout of *Mll2* after E11.5 produced mice without noticeable pathologies suggesting *Mll2* is not required for late development, stem cells or homeostasis in somatic cells, although male mice lacking *Mll2* are infertile. We observed altered mRNA levels of a number of genes shown to be downregulated in these studies [37].

*Neurod1*, downregulated in mutant islets, is required for the regulation of β-cell genes including Insulin, Sulphonylurea Receptor (*Sur1*), Glucagon, Somatasatin and Neuronatin (*Nnat*). *Ins1* was moderately upregulated in islets, probably as a result of the insulin resistance leading to hyperinsulinaemia. *Sur1* a component of the K_ATP_ channel essential for glucose stimulated insulin secretion was unchanged. Somatostatin was upregulated and it has been proposed to exert an inhibitory effect on insulin and glucagon secretion, and may contribute to the insulin dysregulation, although its physiological significance in these roles is unclear (see for example [41], [42]). Glucagon stimulates glucose mobilization and its downregulation may reflect the hyperglycaemia.

The greatest difference observed was reduced *Nnat* gene expression. *Nnat* is an imprinted gene expressed from the paternal allele and CHIP assays suggest that *Nnat* is a direct target of *Neurod1* [43]. Increased expression of *Nnat* results in increased insulin secretion upon acute glucose stimulation and knockdown of *Nnat* in insulin secreting cell lines resulted in a loss of glucose stimulated insulin secretion [43], [44]. The decrease in *Nnat* expression may explain the reduction in glucose stimulated insulin secretion that we observed. *Nnat* is also abundantly expressed in adipose tissue and has a role in the potentiation of adipocyte differentiation [45]. *Nnat* potentiates adipogenesis through enhanced phosphorylation of cAMP-response element–binding protein in 3T3-L1 cells [46], and is upregulated in Zucker diabetic rats compared to control lean Zucker rats [47]. It has also been associated with severe childhood and adult obesity in humans [48]. *Nnat* expression was significantly downregulated in epididymal fat from *Mll2* mutant animals and may lead to reduced adipogenesis and a reduction in mature adipocyes reflected in the reduced fat pad mass observed.

*Slc27a2* (FATP2) is one of the 2 main fatty acid transporters in the liver, with knockdown of expression shown to reduce long chain fatty acid (LCFA) uptake by 40% [49]. This gene was strikingly reduced in expression in islets and adipose tissue. Interestingly, as reduction of FATP2 has been shown to protect against hepatosteatosis on a high fat diet, there was no difference in liver expression between mutant and wild-type and this may predispose to the steatohepatitis that we observed [49]. Why loss of Mll2 function does not lead to a reduction of Slc27a2 expression in liver but does reduce expression in islets and fat and indeed in ES cells [37] is unclear.

Both *Enpp3* and *Enpp1* have both been associated with diabetes. ENPP3 protein was detected in rat pancreas and liver and downregulated in islets by high compared to low glucose in diabetic GK rats, consistent with our observations [50]. Variants of *ENPP1* in human GWAS studies have been associated with obesity, type 2 diabetes and a primary role in insulin resistance [51]. Increased expression of *Enpp1* or its overactivity is correlated with insulin resistance, through direct effects in the insulin signaling pathway. This may explain, at least in the case of increase *Enpp1* expression in skeletal muscle, some of the insulin resistance in our mutant mice (reviewed [52]).

Phospholipases are responsible for the hydrolysis of phosphatidylinositol 4-5-biphosphate (PIP2) to inositol 1,4,5-triphosphate (IP3) and 1,2-diacylglycerol (DAG), both of which have important second messenger functions. Phosphatidylinositol-specific phospholipase C, X domain containing 1 (*Plcxd1*) was shown to be significantly downregulated in all tissues examined. There is limited information about the function of this gene but reduced expression may reflect dysregulation of insulin signalling pathways either through effects on IP3 or DAG.

Type 2 Diabetes is a complex disease involving many different tissue types, euglycemic hyperinsulinemic CLAMP studies may further dissect the tissues important for the insulin resistance identified in this model. CHIP studies in relevant tissues may identify other yet unidentified genes whose regulation by MLL2 at the chromatin level may contribute to disease. It would be informative to carry out these studies before and after onset of overt disease to differentiate between causal and effect differences in gene expression. Whilst the mutation identified is in a single gene Mll2 the phenotype observed is likely to be the result of multiple small changes in the expression of a number of genes in many diverse tissues. Tissue specific KO of MLL2 followed by CHIP analysis and comprehensive metabolic phenotyping should yield insight into the relative contribution of each tissue and gene set to onset of disease.

## Conclusions

In summary, we have identified two ENU induced point mutations M2628K and F2628I in MLL2 that give rise to a novel murine model of insulin resistance, impaired glucose tolerance and primary stages of NAFLD. We have provided evidence that these are functional mutations that affect the H3K4 methyltransferase activity of MLL2 that then leads to changes during embryonic development, likely in chromatin and DNA methylation (see [37]), that determine the expression of genes linked to diabetes phenotypes in the adult. These data reveal that gene expression controlled through histone methylation is a significant mechanism involved in glucose homeostasis.

## Supporting Information

Figure S1 **Sequence alignment of the highly conserved SET domain of MLL1 and MLL2.**
(TIF)

Figure S2 ***In vitro*** **methyltransferase assays indicate a reduced activity of the MLL1 (M3884K) mutant in comparison to wild-type MLL1.** The methylation of histone H3 substrates comprising the first 21 N-terminal amino acids was quantified enzyme linked immunoabsorbent assays (ELISAs) with antibodies against H3K4me1, me2 and me3. ***A***: Unmodified H3 peptide (H3K4me0) was incubated with wild-type and mutant recombinant expressed SET-domain of MLL1 respectively and H3K4me1 product detected (see Figure S3 for time courses of the subsequent products H3K4me2 and H3K4me3). ***B***: Monomethylated H3 peptide (H3K4me1) was incubated with MLL and samples were analyzed for dimethylation (forming H3K4me2 - Figure S3 for time courses of the subsequent product H3K4me3). ***C***: Dimethylated H3 peptide (H3K4me2) was incubated with MLL and H3K4me3 product detected. The activity of the M3884K was reduced compared to wild-type in all cases. The M3884K position of MLL1 is equivalent to the M2628K position of Mll2 based on sequence alignment. Error bars show the SD from the mean value of three experiments.
(TIF)

Figure S3 ***In vitro*** **methyltransferase assays indicate a reduced activity of the MLL1 (M3884K) mutant in comparison to wild-type MLL1.** The methylation of histone H3 substrates comprising the first 21 N-terminal amino acids was quantified enzyme linked immunoabsorbent assays with antibodies against H3K4me1, me2 and me3. **A–C** Unmodified H3 peptide was incubated with wildtype and mutant recombinant expressed SET-domain of MLL1 respectively. Samples were analyzed for (**A**) monomethylation, (**B**) dimethylation and (**C**) trimethylation at lysine 4 after 2, 8, 20 and 48 hours. **D, E** H3 peptide with a monomethyl modified lysine 4 was incubated with the enzyme and the mutant. The samples were analyzed for (**D**) dimethylation and (**E**) trimethylation. (**F**) Lysine 4 dimethyl H3 peptide was incubated with the wild-type enzyme and mutant and samples analyzed for a trimethyl mark at lysine 4. Error bars show the SD from the mean value.
(TIF)

Figure S4 **Adult Knockout of Mll2.** All genotype classes and treatment groups. N = 7–11 for each group. **A**: Plasma glucose measured in an intraperitoneal glucose tolerance test in male mice at 12 weeks of age, **B**: Weight at 12 weeks of age. **C**: Fasted plasma insulin at 12 weeks of age. Data represented as Mean ±SEM.
(TIF)

Figure S5 **Dexa analysis of** ***Mll2^M2628K/+^*** **compared to wildtype litter mates.** Dexa analysis at 8, 12 and 18weeks *Mll2^M2628K/+^* (open bars N = 12) compared to wildtype littermates (Black bars N = 17). No significant difference was observed in total body weight or lean mass at any of the 3 time points. A transient significant increase in body fat in *Mll2^M2628K/+^* was observed at 12 weeks of age (p = 0.03). Data represented as Mean ±SEM.
(TIF)

Figure S6 **Relative expression of Neurod1 regulated genes in Isolated Islets.** Data represents 8 biological replicates, *Mll2^M2628K^*/+ (open bars) vs wt littermates (black bars), data normalized to GAPDH. Relative expression ±SEM * p<0.05, **p<0.01, ***p<0.001, student's t-test.
(TIF)

Table S1 **Candidate list on chromosome 7.**
(TIF)

Table S2 **Percentage Islet areas.**
(TIF)
