# Supplementary figures and images for: Mutations in Mll2, an H3K4 Methyltransferase, Result in Insulin Resistance and Impaired Glucose Tolerance in Mice
Source: PLoS One. 2013 Jun 24;8(6):e61870. doi: 10.1371/journal.pone.0061870 (PMC3691224; doi:10.1371/journal.pone.0061870)

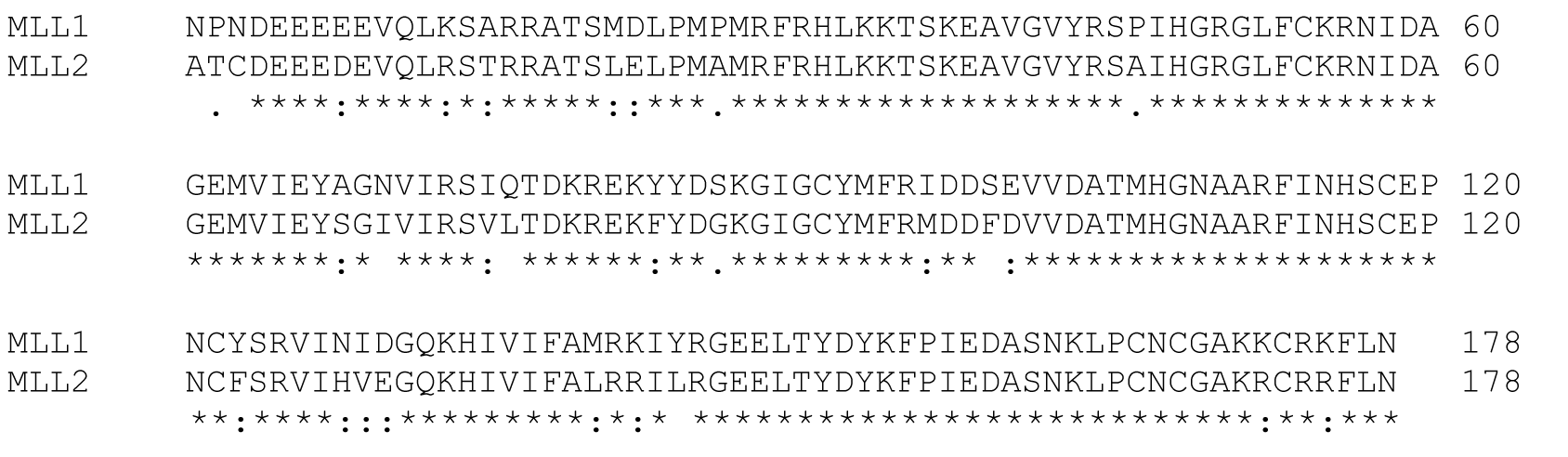

Supplement: Figure S1 — Sequence alignment of the highly conserved SET domain of MLL1 and MLL2. (TIF) [file pone.0061870.s001.tif]

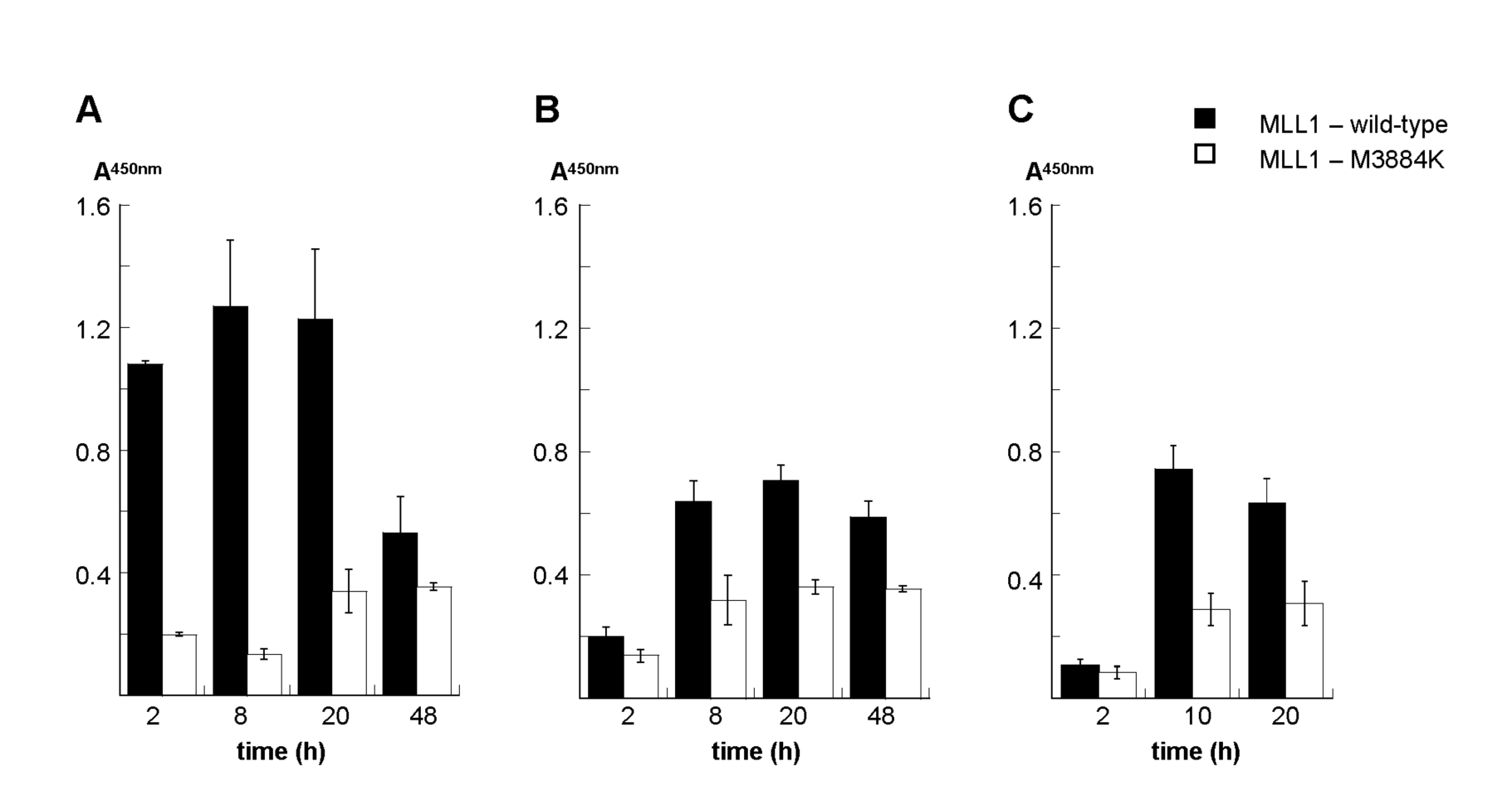

Supplement: Figure S2 — In vitro methyltransferase assays indicate a reduced activity of the MLL1 (M3884K) mutant in comparison to wild-type MLL1. The methylation of histone H3 substrates comprising the first 21 N-terminal amino acids was quantified enzyme linked immunoabsorbent assays (ELISAs) with antibodies against H3K4me1, me2 and me3. A: Unmodified H3 peptide (H3K4me0) was incubated with wild-type and mutant recombinant expressed SET-domain of MLL1 respectively and H3K4me1 product detected (see Figure S3 for time courses of the subsequent products H3K4me2 and H3K4me3). B: Monomethylated H3 peptide (H3K4me1) was incubated with MLL and samples were analyzed for dimethylation (forming H3K4me2 - Figure S3 for time courses of the subsequent product H3K4me3). C: Dimethylated H3 peptide (H3K4me2) was incubated with MLL and H3K4me3 product detected. The activity of the M3884K was reduced compared to wild-type in all cases. The M3884K position of MLL1 is equivalent to the M2628K position of Mll2 based on sequence alignment. Error bars show the SD from the mean value of three experiments. (TIF) [file pone.0061870.s002.tif]

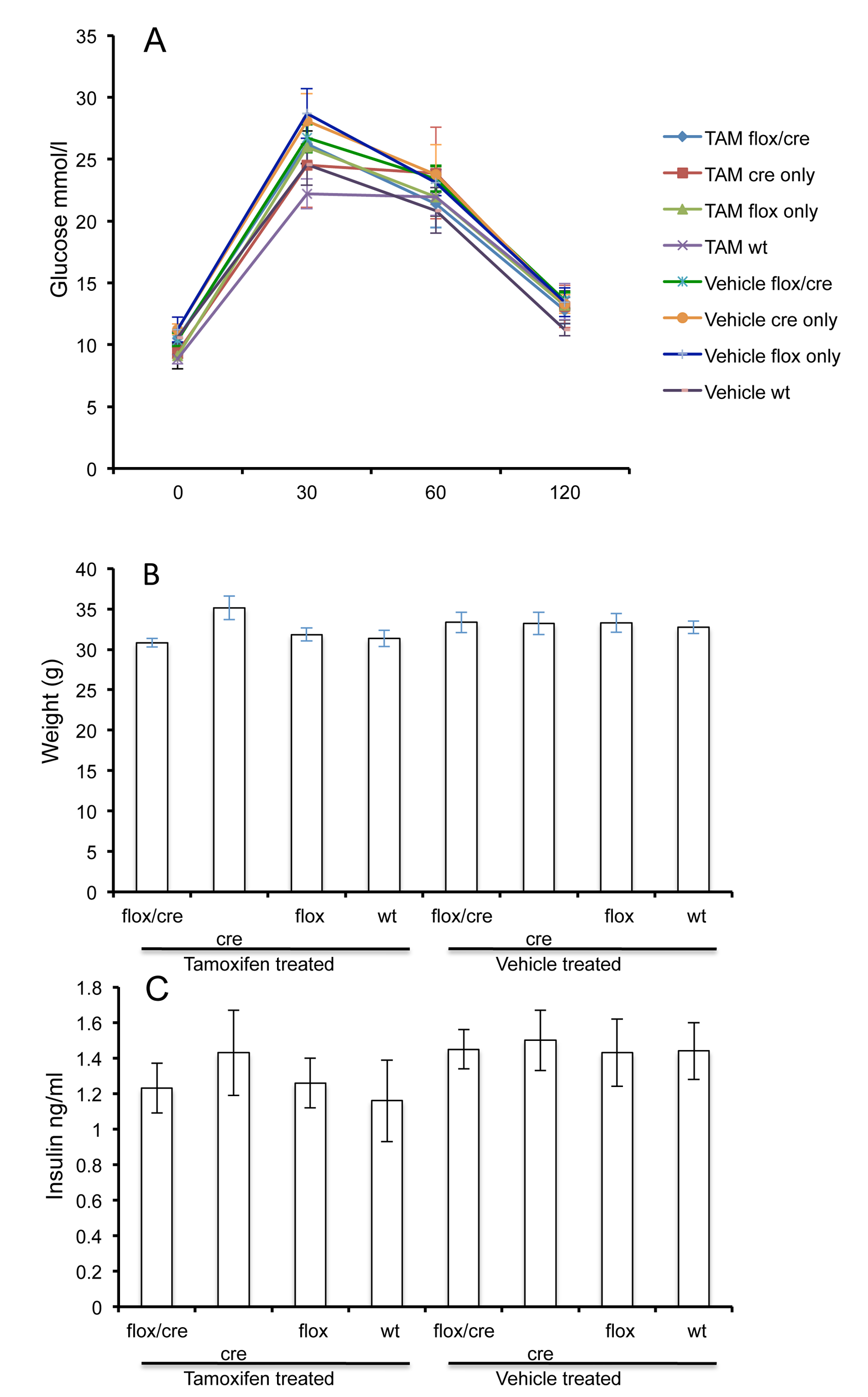

Supplement: Figure S4 — Adult Knockout of Mll2. All genotype classes and treatment groups. N = 7–11 for each group. A: Plasma glucose measured in an intraperitoneal glucose tolerance test in male mice at 12 weeks of age, B: Weight at 12 weeks of age. C: Fasted plasma insulin at 12 weeks of age. Data represented as Mean ±SEM. (TIF) [file pone.0061870.s004.tif]

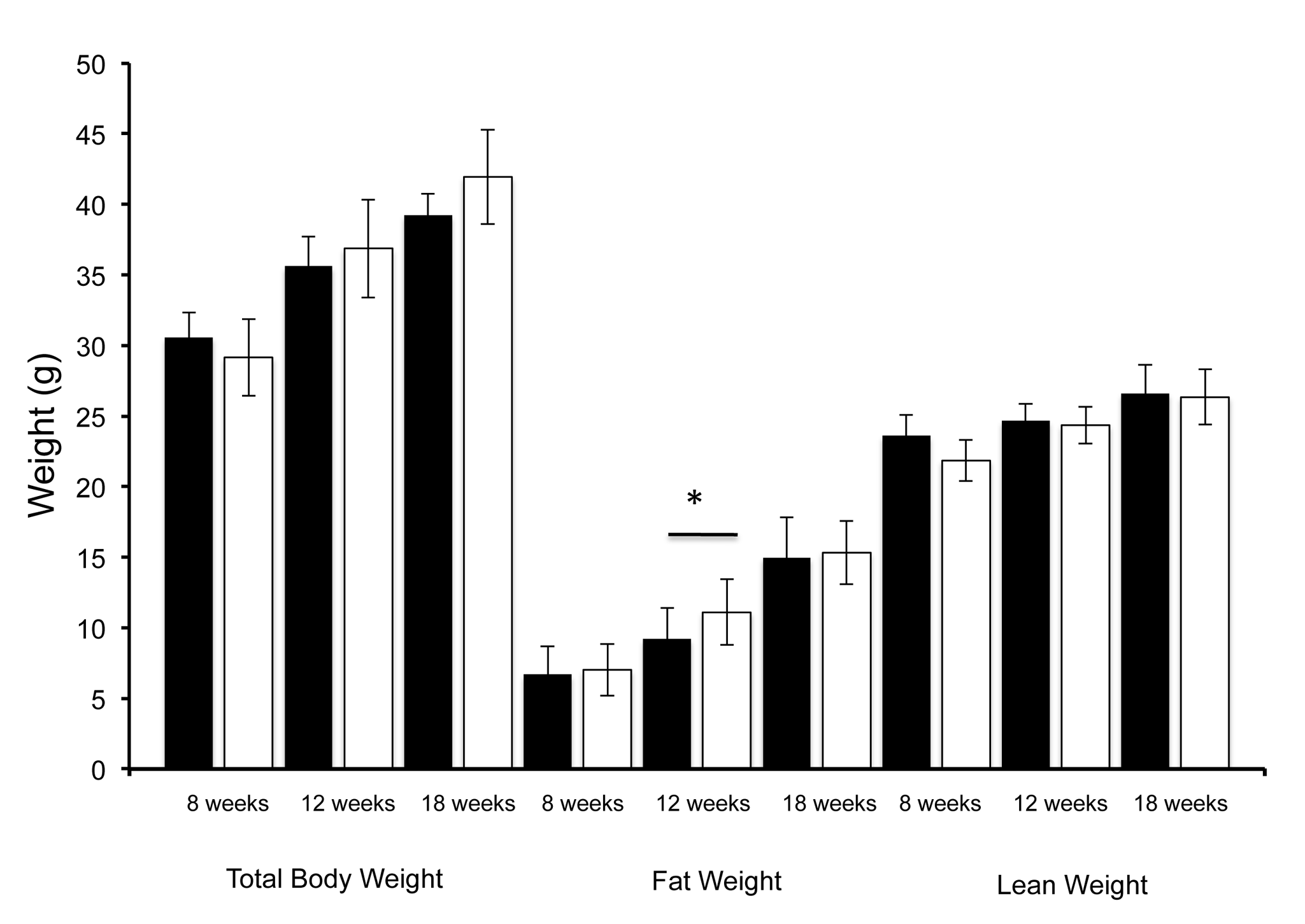

Supplement: Figure S5 — Dexa analysis of Mll2M2628K/+ compared to wildtype litter mates. Dexa analysis at 8, 12 and 18weeks Mll2M2628K/+ (open bars N = 12) compared to wildtype littermates (Black bars N = 17). No significant difference was observed in total body weight or lean mass at any of the 3 time points. A transient significant increase in body fat in Mll2M2628K/+ was observed at 12 weeks of age (p = 0.03). Data represented as Mean ±SEM. (TIF) [file pone.0061870.s005.tif]

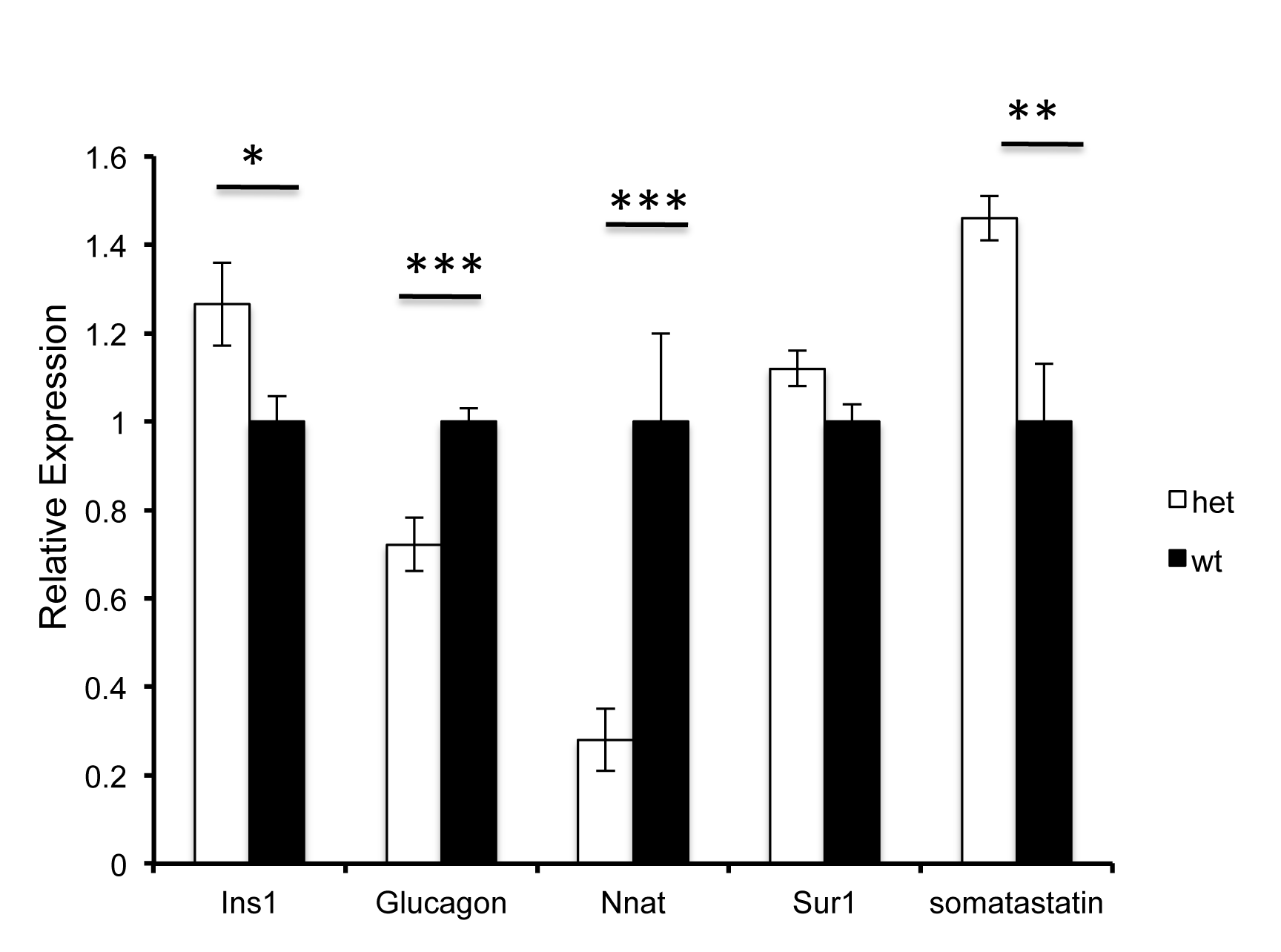

Supplement: Figure S6 — Relative expression of Neurod1 regulated genes in Isolated Islets. Data represents 8 biological replicates, Mll2M2628K/+ (open bars) vs wt littermates (black bars), data normalized to GAPDH. Relative expression ±SEM * p<0.05, **p<0.01, ***p<0.001, student's t-test. (TIF) [file pone.0061870.s006.tif]

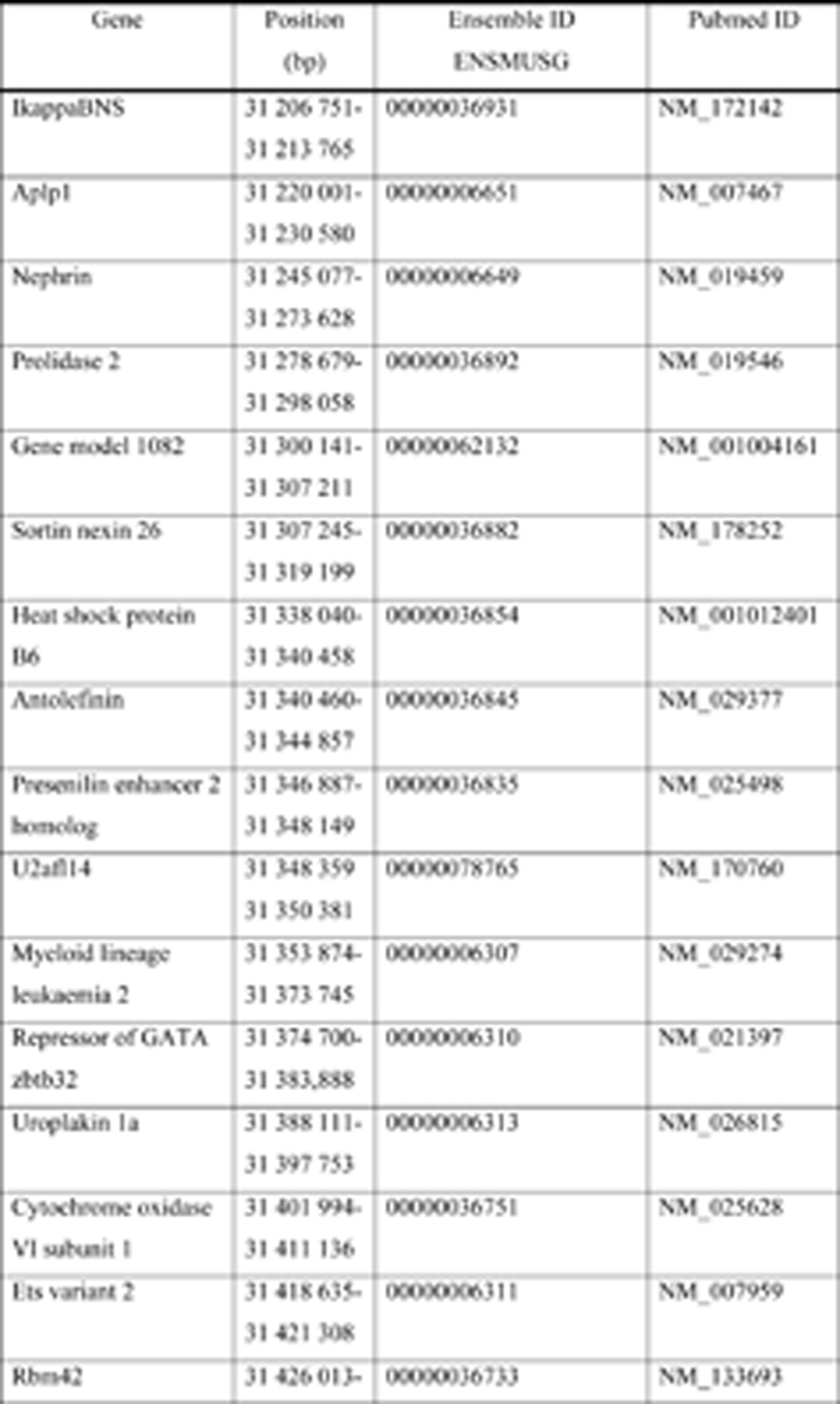

Supplement: Table S1 — Candidate list on chromosome 7. (TIF) [file pone.0061870.s007.tif]

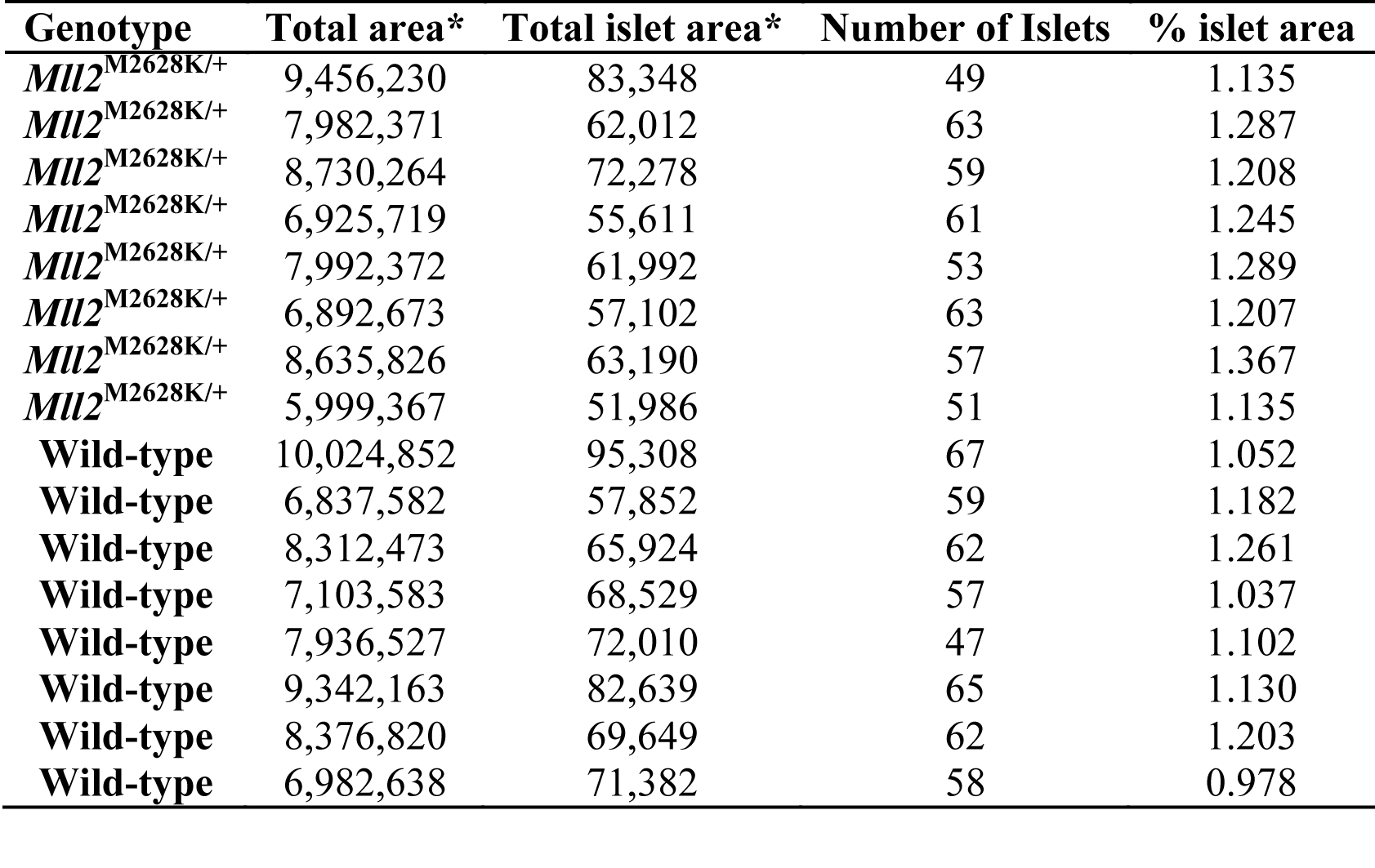

Supplement: Table S2 — Percentage Islet areas. (TIF) [file pone.0061870.s008.tif]
